# Occupational Patterns in Unintentional and Undetermined Drug-Involved and Opioid-Involved Overdose Deaths — United States, 2007–2012

**DOI:** 10.15585/mmwr.mm6733a3

**Published:** 2018-08-24

**Authors:** Laurel Harduar Morano, Andrea L. Steege, Sara E. Luckhaupt

**Affiliations:** ^1^Epidemic Intelligence Service, CDC; ^2^Division of Surveillance, Hazard Evaluations and Field Studies, National Institute for Occupational Safety and Health, CDC.

## Abstract

The opioid epidemic affects multiple segments of the U.S. population (*1*). Occupational patterns might be critical to understanding the epidemic. Opioids are often prescribed for specific types of work-related injuries, which vary by occupation* (*2*). CDC used mortality data from the National Occupational Mortality Surveillance (NOMS) system to examine unintentional or undetermined drug overdose mortality within 26 occupation groups. This study included data from the 21 U.S. states participating in NOMS during 2007–2012.^†^ Drug overdose mortality was compared with total mortality using proportional mortality ratios (PMRs) indirectly standardized for age, sex, race, year, and state. Mortality patterns specific to opioid-related overdose deaths were also assessed. Construction occupations had the highest PMRs for drug overdose deaths and for both heroin-related and prescription opioid–related overdose deaths. The occupation groups with the highest PMRs from methadone, natural and semisynthetic opioids, and synthetic opioids other than methadone were construction, extraction (e.g., mining, oil and gas extraction), and health care practitioners. The workplace is an integral part of life for the majority of the adult U.S. population; incorporating workplace research and interventions likely will benefit the opioid epidemic response.

NOMS is a population-based surveillance system and a collaborative effort between state vital statistics offices and CDC’s National Institute for Occupational Safety and Health (NIOSH) and National Center for Health Statistics (NCHS). Through data sharing agreements with NIOSH, all participating states, or NCHS under states’ direction, share selected data from their death certificates, including the decedent’s usual industry and occupation, coded to the U.S. Census industry and occupation codes. This analysis includes 4,024,086 deaths that occurred in persons aged ≥18 years, from the 21 states that contributed ≥1 year of data^†^ to NOMS during 2007–2012.^§^

*International Classification of Disease, Tenth Revision* (ICD-10) codes for underlying cause of death were used to identify unintentional (X40–X44) and undetermined (Y10–Y14) drug overdose deaths. Among drug overdose deaths, the specific type of opioid was indicated by the following ICD-10 multiple cause of deaths codes: T40.1 (heroin) and T40.2–T40.4 (prescription opioids [i.e., T40.2, natural and semisynthetic opioids; T40.3, methadone; and T40.4, synthetic opioids other than methadone]).^¶^ Deaths that involved multiple opioid types were included in multiple categories.

Usual occupation, recorded as free-text on the death certificate, was coded to 1990 or 2000 U.S. Census occupation codes** by NIOSH or by the state. A crosswalk based on U.S. Census data was used to convert the 1990 U.S. Census occupation codes to the 2000 U.S. Census occupation codes.^††^ Occupation codes were binned into 26 groups based on job duties.^§§^ For each outcome, the proportion of deaths among each occupation group was compared with the proportion of deaths among all occupations combined using PMRs indirectly standardized by age, sex, race, calendar year, and state of occurrence. A PMR >1.00 indicated that the proportion of deaths within that occupation group is higher than the proportion of deaths among all occupation groups combined. Corresponding 95% confidence intervals were calculated.

The analysis identified 57,810 drug overdose deaths within the study population (1.4% of the 4,024,086 deaths). The majority of drug overdose deaths were among persons who were male (61.8%), white (89.8%), and aged 45–54 years (30.1%) or 35–44 years (24.1%).^¶¶^ PMRs from drug overdose were significantly above 1.00 for the following six occupation groups: 1) construction (1.25); 2) extraction (1.16); 3) food preparation and serving (1.11); 4) health care practitioners and technical (1.16); 5) health care support (1.18); and 6) personal care and service (1.10) (Table 1). PMRs from drug overdose were also significantly elevated among deaths where the usual occupation was unpaid/unemployed (1.10)*** or unknown (1.31).^†††^ For each specific opioid type, significantly elevated PMRs were generally observed only for those occupation groups that also had a significantly elevated PMR for drug overdose overall (Table 1) (Table 2). The only two exceptions were the arts, design, entertainment, sports, and media occupation group and the building and grounds cleaning and maintenance occupation group. For these groups, the proportion of drug overdose deaths among the two occupation groups was similar to the proportion of drug overdose deaths overall (i.e., PMR approximately = 1.00), whereas the proportional distribution of specific drugs involved in an overdose was different,^§§§^ with heroin-involved overdose deaths higher than expected (Table 1). The highest PMRs for methadone, natural and semisynthetic opioids, and synthetic opioids were in the construction (1.34), extraction (1.39), and healthcare practitioner (1.81) occupation groups, respectively.

**TABLE 1:** Usual occupation group and mortality from unintentional or undetermined drug overdoses* and drug overdoses involving heroin^†^ or opioid analgesics^§^ — National Occupational Mortality Surveillance, United States, 2007–2012

U.S. Census 2000 occupation group^¶^	Total no. of deaths observed	Drug overdose*			Heroin^†^			Prescription opioid^§^		
		Deaths		Standardized PMR (95% CI)**	Deaths		Standardized PMR (95% CI)**	Deaths		Standardized PMR (95% CI)**
		No. observed	No. expected		No. observed	No. expected		No. observed	No. expected	
**Total**	**4,024,086**	**57,810**	**—^††^**	**—^††^**	**7,463**	**—^††^**	**—^††^**	**25,058**	**—^††^**	**—^††^**
Management	325,123	2,458	3,324.2	0.74 (0.71–0.77)	232	383.2	0.61 (0.53–0.69)	1,106	1,446.7	0.76 (0.72–0.81)
Business operations	38,740	349	496.2	0.70 (0.63–0.78)	31	51.2	0.61 (0.41–0.86)	155	213.2	0.73 (0.62–0.85)
Financial	51,795	390	575.2	0.68 (0.61–0.75)	31	57.5	0.54 (0.37–0.77)	181	254.6	0.71 (0.61–0.82)
Computer and mathematical	21,425	422	585.2	0.72 (0.65–0.79)	57	85.5	0.67 (0.5–0.86)	187	255.6	0.73 (0.63–0.84)
Architecture and engineering	88,825	580	839.3	0.69 (0.64–0.75)	62	116.5	0.53 (0.41–0.68)	265	354.1	0.75 (0.66–0.84)
Life, physical, and social science	24,332	257	301.8	0.85 (0.75–0.96)	20	37.9	0.53 (0.32–0.81)	124	133.8	0.93 (0.77–1.11)
Community and social services	39,046	381	449.3	0.85 (0.77–0.94)	35	48.6	0.72 (0.50–1.00)	160	190.4	0.84 (0.72–0.98)
Legal	17,677	208	254.3	0.82 (0.71–0.94)	15	24.1	0.62 (0.35–1.03)	98	116.1	0.84 (0.69–1.03)
Education, training, and library	146,334	701	1,187.8	0.59 (0.55–0.64)	46	109.1	0.42 (0.31–0.56)	289	514.8	0.56 (0.50–0.63)
Arts, design, entertainment, sports, and media	48,331	929	898.7	1.03 (0.97–1.10)	144	119.4	1.21 (1.02–1.42)	412	401.0	1.03 (0.93–1.13)
Health care practitioners and technical^††^	126,901	1,839	1,592.0	1.16 (1.10–1.21)	109	139.3	0.78 (0.64–0.94)	876	709.2	1.24 (1.15–1.32)
Health care support^§§^	57,196	1,363	1,153.1	1.18 (1.12–1.25)	116	106.7	1.09 (0.90–1.30)	626	518.9	1.21 (1.11–1.30)
Protective service	57,986	653	909.7	0.72 (0.66–0.78)	64	125.6	0.51 (0.39–0.65)	299	382.7	0.78 (0.7–0.88)
Food preparation and serving^§§^	109,961	2,885	2,595.3	1.11 (1.07–1.15)	486	345.6	1.41 (1.28–1.54)	1,207	1,142.6	1.06 (1.00–1.12)
Building and grounds cleaning and maintenance	121,966	2,025	2,090.4	0.97 (0.93–1.01)	344	294.7	1.17 (1.05–1.30)	811	888.9	0.91 (0.85–0.98)
Personal care and service^§§^	67,288	1,333	1,207.3	1.10 (1.05–1.17)	144	125.4	1.15 (0.97–1.35)	612	540.3	1.13 (1.04–1.23)
Sales	287,191	3,413	3,795.9	0.90 (0.87–0.93)	405	460.4	0.88 (0.80–0.97)	1,515	1,684.7	0.90 (0.85–0.95)
Office and administrative support	345,607	2,861	3,523.8	0.81 (0.78–0.84)	261	346.8	0.75 (0.66–0.85)	1,341	1,564.7	0.86 (0.81–0.90)
Farming, fishing, and forestry	27,421	354	482.4	0.73 (0.66–0.81)	49	66.1	0.74 (0.55–0.98)	158	222.1	0.71 (0.60–0.83)
Construction^§§^	244,534	7,402	5,902.5	1.25 (1.23–1.28)	1,345	922.9	1.46 (1.38–1.54)	3,122	2,573.0	1.21 (1.17–1.26)
Extraction^§§^	19,536	431	370.8	1.16 (1.06–1.28)	35	43.9	0.80 (0.55–1.11)	263	201.7	1.30 (1.15–1.47)
Installation, maintenance, and repair	124,578	2,179	2,201.1	0.99 (0.95–1.03)	319	339.5	0.94 (0.84–1.05)	950	945.6	1.00 (0.94–1.07)
Production	370,855	3,662	3,871.5	0.95 (0.92–0.98)	514	571.6	0.90 (0.82–0.98)	1,544	1,580.7	0.98 (0.93–1.03)
Transportation and material moving	276,558	4,370	4,656.7	0.94 (0.91–0.97)	710	721.1	0.98 (0.91–1.06)	1,680	1,869.1	0.90 (0.86–0.94)
Military specific	37,616	352	425.3	0.83 (0.74–0.92)	41	60.9	0.67 (0.48–0.91)	142	188.5	0.75 (0.63–0.89)
Nonpaid workers^§§^	856,256	13,001	11,819.2	1.10 (1.08–1.12)	1,324	1,380.0	0.96 (0.91–1.01)	5,783	5,250.3	1.10 (1.07–1.13)
Unknown^§§^	91,008	3,012	2,301.4	1.31 (1.26–1.36)	524	379.7	1.38 (1.26–1.50)	1,152	914.8	1.26 (1.19–1.33)

**Abbreviations**: CI = confidence interval; NOMS = National Occupational Mortality Surveillance; PMR = proportionate mortality ratio.

* Deaths were classified using the *International Classification of Diseases, Tenth Revision (*ICD–10*)*. Drug overdose deaths were identified using underlying cause-of death codes X40–X44 (unintentional) and Y10–Y14 (unknown intent).

^†^ Drug overdose deaths, as defined, that have heroin (T40.1) as a contributing cause.

^§^ Drug overdose deaths, as defined, that have prescription opioids (T40.2-T40.4) as a contributing cause.

^¶^ Occupation groups presented in ascending 2000 census code order (e.g., Management = 001–043); https://usa.ipums.org/usa/volii/occ2000.shtml.

** Indirectly standardized to the standard population of all NOMS deaths with occupation information by age, sex, race (white, black, other), calendar year (2007–2012), and state.

^††^ Not applicable.

^§§^ PMR significantly above 1.00 for drug overdose deaths in these categories.

**TABLE 2:** Usual occupation group and mortality from unintentional and undetermined drug overdoses* involving natural and semisynthetic opioids^†^, methadone^§^, or synthetic opioids other than methadone^¶^ — National Occupational Mortality Surveillance, United States, 2007–2012

U.S. Census 2000 occupation group**	Total no. of deaths observed	Natural and semisynthetic opioids*			Methadone^†^			Synthetic opioids other than methadone^§^		
		Deaths		Standardized PMR (95% CI)^††^	Deaths		Standardized PMR (95% CI)^††^	Deaths		Standardized PMR (95% CI)^††^
		No. observed	No. expected		No. observed	No. expected		No. observed	No. expected	
**Total**	**4,024,086**	**16,603**	**—^§§^**	**—^§§^**	**7,504**	**—^§§^**	**—^§§^**	**3,966**	**—^§§^**	**—^§§^**
Management	325,123	747	965.5	0.77 (0.72–0.83)	326	433.0	0.75 (0.67–0.84)	177	223.7	0.79 (0.68–0.92)
Business operations	38,740	111	139.8	0.79 (0.65–0.96)	34	63.8	0.53 (0.37–0.74)	29	36.2	0.80 (0.54–1.15)
Financial	51,795	132	170.6	0.77 (0.65–0.92)	40	74.3	0.54 (0.38–0.73)	28	41.9	0.67 (0.44–0.97)
Computer and mathematical	21,425	120	167.1	0.72 (0.60–0.86)	51	81.2	0.63 (0.47–0.83)	35	37.4	0.94 (0.65–1.30)
Architecture and engineering	88,825	178	230.3	0.77 (0.66–0.90)	75	114.0	0.66 (0.52–0.82)	32	51.6	0.62 (0.42–0.88)
Life, physical, and social science	24,332	85	88.0	0.97 (0.77–1.19)	31	43.1	0.72 (0.49–1.02)	22	18.7	1.18 (0.74–1.78)
Community and social services	39,046	100	126.8	0.79 (0.64–0.96)	46	55.7	0.83 (0.60–1.10)	34	31.3	1.09 (0.75–1.52)
Legal	17,677	73	78.2	0.93 (0.73–1.17)	18	34.2	0.53 (0.31–0.83)	18	18.4	0.98 (0.58–1.54)
Education, training, and library	146,334	215	346.4	0.62 (0.54–0.71)	50	143.6	0.35 (0.26–0.46)	65	89.0	0.73 (0.56–0.93)
Arts, design, entertainment, sports, and media	48,331	268	264.8	1.01 (0.89–1.14)	125	124.5	1.00 (0.84–1.20)	60	57.9	1.04 (0.79–1.33)
Health care practitioners and technical	126,901	565	474.5	1.19 (1.09–1.29)	199	198.1	1.00 (0.87–1.15)	229	126.3	1.81 (1.59–2.06)
Health care support	57,196	396	339.5	1.17 (1.05–1.29)	197	152.4	1.29 (1.12–1.49)	106	93.4	1.13 (0.93–1.37)
Protective service	57,986	216	257.0	0.84 (0.73–0.96)	76	115.9	0.66 (0.52–0.82)	49	55.0	0.89 (0.66–1.18)
Food preparation and serving	109,961	765	744.9	1.03 (0.96–1.10)	400	357.3	1.12 (1.01–1.23)	180	176.3	1.02 (0.88–1.18)
Building and grounds cleaning and maintenance	121,966	544	591.7	0.92 (0.84–1.00)	249	265.4	0.94 (0.83–1.06)	119	137.3	0.87 (0.72–1.04)
Personal care and service	67,288	411	361.1	1.14 (1.03–1.25)	205	159.0	1.29 (1.12–1.48)	89	87.5	1.02 (0.82–1.25)
Sales	287,191	1,039	1,118.5	0.93 (0.87–0.99)	422	507.2	0.83 (0.75–0.92)	229	261.5	0.88 (0.77–1.00)
Office and administrative support	345,607	908	1,042.3	0.87 (0.82–0.93)	366	450.2	0.81 (0.73–0.90)	233	269.0	0.87 (0.76–0.98)
Farming, fishing, and forestry	27,421	103	140.0	0.74 (0.60–0.89)	54	80.5	0.67 (0.50–0.87)	23	28.4	0.81 (0.51–1.22)
Construction	244,534	2,013	1,696.2	1.19 (1.14–1.24)	1,075	805.2	1.34 (1.26–1.42)	416	366.3	1.14 (1.03–1.25)
Extraction	19,536	208	149.7	1.39 (1.21–1.59)	42	45.8	0.92 (0.66–1.24)	41	33.2	1.23 (0.89–1.67)
Installation, maintenance, and repair	124,578	631	625.8	1.01 (0.93–1.09)	304	293.2	1.04 (0.92–1.16)	132	135.0	0.98 (0.82–1.16)
Production	370,855	1,018	1,034.0	0.98 (0.93–1.05)	470	477.7	0.98 (0.90–1.08)	237	256.6	0.92 (0.81–1.05)
Transportation and material moving	276,558	1,084	1,227.8	0.88 (0.83–0.94)	548	572.6	0.96 (0.88–1.04)	235	283.2	0.83 (0.73–0.94)
Military specific	37,616	87	122.3	0.71 (0.57–0.88)	41	62.8	0.65 (0.47–0.89)	28	23.5	1.19 (0.79–1.72)
Nonpaid workers	856,256	3,841	3,485.7	1.10 (1.07–1.14)	1,705	1,527.7	1.12 (1.06–1.17)	946	887.7	1.07 (1.00–1.14)
Unknown	91,008	745	614.6	1.21 (1.13–1.30)	355	265.7	1.34 (1.20–1.48)	174	139.7	1.25 (1.07–1.44)

**Abbreviations:** CI = confidence interval; NOMS = National Occupational Mortality Surveillance; PMR = proportionate mortality ratio.

* Deaths were classified using the *International Classification of Diseases, Tenth Revision (*ICD–10*)*. Drug overdose deaths were identified using underlying cause-of death codes X40–X44 (unintentional) and Y10–Y14 (unknown intent).

^†^ Drug overdose deaths, as defined, with natural and semisynthetic opioids (T40.2) as a contributing cause.

^§^ Drug overdose deaths, as defined, with methadone (T40.3) as a contributing cause.

^¶^ Drug overdose deaths, as defined, with synthetic opioids other than methadone (T40.4) as a contributing cause. This category includes legal and illegal fentanyl along with other synthetic opioids.

** Occupation groups presented in ascending 2000 census code order (e.g., Management = 001–043); https://usa.ipums.org/usa/volii/occ2000.shtml.

^††^ Indirectly standardized to the standard population of all NOMS deaths with occupation information by age, sex, race (white, black, other), calendar year (2007–2012), and state.

^§§^ Not applicable.

Because the PMRs for all opioid types within the construction occupation group were elevated, a subanalysis further examined opioid-related deaths in this group. The analysis identified 7,402 drug overdose deaths among persons aged ≥18 years within the construction occupation group. The majority of decedents were male (96.7%), white (92.6%), and aged 45–54 years (30.4%) or 35–44 years (26.9%).^¶¶¶^ These deaths were examined by the following occupation subgroups****: first-line supervisors and managers,^††††^ construction trade workers (e.g., carpenters, electricians, painters, iron and steel workers, operating engineers, and construction equipment operators), construction trade helpers, and other construction and related workers (e.g., building inspectors, hazardous waste workers, and highway maintenance workers). PMRs were significantly elevated for all types of opioids within the occupation subgroup construction trade workers (Table 3).

**TABLE 3:** Construction occupation subgroup* and mortality from unintentional and undetermined drug overdoses^†^ by drug type — National Occupational Mortality Surveillance, United States, 2007–2012

Opioid type	First-line supervisors/managers		Construction trades workers		Helpers, construction		Other construction and related workers	
	No. observed	PMR (95% CI)^§^	No. observed	PMR (95% CI)^§^	No. observed	PMR (95% CI)^§^	No. observed	PMR (95% CI)^§^
**Total**	**24,306**	**—^¶^**	**213,029**	**—^¶^**	**419**	**—^¶^**	**6,780**	**—^¶^**
Overdose	338	0.94 (0.84–1.05)	6,901	1.28 (1.25–1.31)	26	1.31 (0.85–1.91)	137	1.15 (0.97–1.36)
Heroin**	44	0.88 (0.64–1.18)	1,282	1.51 (1.42–1.59)	—^††^	—**^††^**	15	0.84 (0.47–1.38)
Prescription opioids^§§^	148	0.96 (0.81–1.12)	2,911	1.23 (1.19–1.28)	12	1.42 (0.73–2.48)	51	1.00 (0.74–1.31)
Natural semisynthetic^¶¶^	92	0.9 (0.72–1.10)	1,876	1.21 (1.15–1.26)	6	1.07 (0.39–2.32)	39	1.16 (0.82–1.58)
Methadone***	49	1.01 (0.75–1.33)	1,007	1.36 (1.28–1.45)	—^††^	—^††^	15	0.93 (0.52–1.53)
Synthetic^†††^	27	1.26 (0.83–1.83)	383	1.14 (1.03–1.26)	—^††^	—^††^	—^††^	—^††^

**Abbreviations:** CI = confidence interval; NOMS = National Occupational Mortality Surveillance; PMR = proportionate mortality ratio.

* Construction first-line supervisors and managers = census 2000 occupation code 620; construction trade workers = census 2000 occupation codes 621–653; construction trade helpers = census 2000 occupation code 660; other construction and related workers = census 2000 occupation codes 666–676.

^†^ Deaths were classified using *International Classification of Diseases, Tenth Revision* (ICD–10*)*. Drug overdose deaths were identified using underlying cause-of death codes X40–X44 (unintentional) and Y10–Y14 (unknown intent).

^§^ Indirectly standardized to the standard population of all NOMS deaths with occupation information by age, sex, race (white, black, other), calendar year (2007–2012), and state.

^¶^ Not applicable.

** Drug overdose deaths, as defined, that have heroin (T40.1) as a contributing cause.

^††^ Observations <5 are not shown. PMRs were not calculated.

^§§^ Drug overdose deaths, as defined, that have prescription opioids (T40.2-T40.4) as a contributing cause.

^¶¶^ Drug overdose deaths, as defined, that have natural and semisynthetic opioids (T40.2) as a contributing cause.

*** Drug overdose deaths, as defined, that have methadone (T40.3) as a contributing cause.

^†††^ Drug overdose death, as defined, that have synthetic opioids other than methadone (T40.4) as a contributing cause. This category includes legal and illegal fentanyl along with other synthetic opioids.

## Discussion

In this study, unintentional and undetermined overdose deaths varied by occupation group, with the construction group having elevated PMRs for all drug types. Although few related studies have been conducted, similar results have been observed. In Kentucky (2011) (*3*) and Ohio (2016) (*4*), for example, overdose deaths varied by industry and occupation and were highest among construction workers. Multiyear studies conducted in two Massachusetts jurisdictions (Barnstable County and Mystic Valley Public Health Coalition communities) found trade workers (e.g., construction, building/grounds maintenance, and mechanics) had the largest proportion of opioid overdose deaths (37% and 42%, respectively) (*5*,*6*). Variation was expected because work-related injuries and illnesses vary by occupation and industry. In addition, other factors that might affect opioid use, such as psychosocial work-related stress (e.g., job insecurity or high demand/low control jobs), socioeconomic standing, and education level, also vary by occupation and industry (*7*–*9*).

The specific drugs influencing higher than expected proportions of overdose deaths also varied by occupation group. In this study, heroin PMRs were highest for the construction; food preparation and serving; and arts, design, entertainment, sports, and media occupation groups. Among the drug types evaluated, heroin is illicit, whereas among the other types, usage is both licit (i.e., prescribed and used as directed) and illicit. Data from the National Survey on Drug Use and Health illustrate that self-reported illicit drug use varies by industry.^§§§§^ The top three industries among persons aged 18–64 years who reported using illicit drugs in the past month were accommodations and food services; arts, entertainment, and recreation; and construction (*10*).

The variation by occupation group in this study leads to speculation about opioid initiation or use and the work environment. A single on-the-job injury (e.g., fracture or dislocation) or chronic work-related pain (e.g., caused by repetitive motion or lifting) might result in a prescription for pain medication (*2*,*8*). Workers’ compensation data from 26 states (2013–2015) indicated that opioids were prescribed for 52%–80% of injured workers who received pain medications (*2*). Persons might also self-medicate or work in an environment with normative support for illicit drug use (*9*). An estimated 64.2% of self-reported illicit opioid^¶¶¶¶^ users were employed full-time or part-time in 2016.***** As licit and illicit opioid users participate in the workforce, occupation might be an important factor in understanding and responding to the opioid epidemic.

The findings in this report are subject to at least six limitations. First, data were analyzed in aggregate, but occupational patterns for each drug type might have differed by year. Second, NOMS has limited information on the specific circumstances of death. It is not known, for example, whether the death occurred at work. Death certificates do not state whether decedents were employed at their usual job (listed on the death certificate), another job, or unemployed at the time of death; if the drug use was legal or illegal; or if drug use was initiated while decedents were employed at their usual job, another job, or before employment. Third, the specific drug involved in the drug overdose death might have been misclassified (e.g., heroin deaths misclassified as morphine deaths because of similar metabolites) or given nonspecific codes (*1*). Within this study, the only drug code listed for one fourth of overdose deaths was “other and unspecified drugs” (T50.9 excluding T36–T50.8). Outcome misclassification might vary by state and year. Fourth, intentional overdose deaths were excluded; however, an unknown proportion of undetermined deaths might have included homicides or suicides and might therefore have resulted in overestimates. In this study, 9.6% of drug overdose deaths were of undetermined intent. The distribution of overdose deaths by intent and occupation group need to be explored. Fifth, PMRs are mutually dependent and cannot distinguish whether occupation was associated with increasing a specific cause of death, preventing the occurrence of other causes of death, or some combination of these effects. Finally, only 21 states participated in NOMS during the study period, which limits generalizability of the findings.

This study identified occupation groups with a higher proportion of drug and opioid-specific overdose mortality but was unable to identify specific factors that might have led to the observed results. The surveillance data presented in this study generated many questions; future studies are needed to identify potential work-related factors along the causal pathway from drug initiation to overdose mortality and to investigate ways of tailoring prevention measures to specific occupations. Workplace-specific programs and policies to reduce the impact of the opioid epidemic can be implemented. Since 2009, a decline in opioid use among nonsurgical workers’ compensation claims in 26 states has occurred, which is associated with changes to workers’ compensation laws and regulations regarding pain management and the prescribing and distribution of opioids, in addition to corresponding national and state-level legislative and regulatory changes (*2*). Examples of programs^†††††^ that might address both licit and illicit opioids include comprehensive drug-free workplace programs, employee assistance programs, peer-support networks, and education targeted to employees and employers (*3*,*5*,*6*). Continued evaluation of the effectiveness and impact of these programs and interventions are needed to prevent opioid misuse and abuse and to reduce opioid-related morbidity and mortality.

**Summary**

**What is already known about this topic?**

A majority of the U.S. population participates in the workforce. A person’s job affects both physical and psychological well-being. The opioid epidemic negatively affects workers, workplaces, and employers.

**What is added by this report?**

During 2007–2012 proportional mortality ratios (PMR) for heroin-related overdose deaths (1.46) and methadone-related overdose deaths (1.34) were highest for the construction occupation group. PMRs for natural and semisynthetic opioids were highest for the extraction (1.39) and health care practitioner (1.81) occupation groups.

**What are the implications for public health practice?**

Identification of occupations associated with drug overdose deaths further characterizes the opioid epidemic. Incorporating workplace research and targeted interventions might benefit the opioid epidemic response.

## Footnotes

* The Bureau of Labor Statistics provides work-related injury, illness, and fatality data by industry and occupation. https://www.bls.gov/iif/.

† Participating states (participation years): Florida (2012), Georgia (2011, 2012), Hawaii (2007–2012), Idaho (2007–2012), Indiana (2007–2010), Kansas (2007–2012), Kentucky (2010–2012), Louisiana (2008–2010), Michigan (2007–2012), Nebraska (2007–2011), Nevada (2007–2012), New Hampshire (2007–2012), New Jersey (2007–2012), New Mexico (2007–2012), North Dakota (2008–2012), Ohio (2007–2012), Texas (2007–2010), Utah (2007–2012), Vermont (2012), Washington (2007–2012), and West Virginia (2007–2012)

§ 2012 is the most recent year for which NOMS data are available. NOMS is the largest source of U.S. population-level mortality data that contains occupation and industry information. Only 21 states contributed data to NOMS during the study period.

¶ From 2013 to 2014, a large increase in illicitly manufactured fentanyl occurred. Within the study period, this category is mostly recording information about pharmaceutical synthetic opioids.

** https://www2.census.gov/programs-surveys/demo/guidance/industry-occupation/occ2000t.pdf. The 1990 census occupation codes are available upon request from the NOMS program (https://www.cdc.gov/niosh/topics/noms/).

†† The crosswalk is based on data in Table 2 of U.S. Census Bureau Technical Paper #65. https://www.census.gov/content/dam/Census/library/working-papers/2003/demo/techpaper2000.pdf.

§§ https://usa.ipums.org/usa/volii/occ2000.shtml.

¶¶ Opioid overdose decedent median age = 43 years.

*** Homemaker (not working on a farm), volunteer, or student.

††† This category includes deaths with insufficient information available on the death certificate to apply a U.S. Census occupation code or for which the usual occupation field was left blank.

§§§ The proportional distribution of drugs (i.e., the proportion of total drug overdoses deaths for each drug type) involved in drug overdose deaths include opioids and nonopioids (e.g., cocaine). PMRs are mutually dependent and a higher proportion for one cause (e.g., a specific drug) results in a lower proportion for another cause. In this analysis, cause-specific outcomes (e.g., heroin-related overdose or prescription opioid–related overdose) are not independent and are partially overlapping. Decedents might have multiple drug types within their system at time of death and therefore counted in more than one cause-specific outcome category.

¶¶¶ Construction occupation group.

**** Construction first-line supervisors and managers = census 2000 occupation code 620; construction trade workers = census 2000 occupation codes 621–653; construction trade helpers = census 2000 occupation code 660; other construction and related workers = census 2000 occupation codes 666–676. https://www.cdc.gov/niosh/topics/coding/pdfs/2000_Census_Occupation.pdf.

†††† This occupation group includes supervisors/managers for both construction and extraction. A subcode to separate construction supervisors/managers from extraction supervisors/managers is not available.

§§§§ Within “Industry,” jobs are organized into categories by type of establishment/business whereas within “Occupation,” jobs are organized into categories with similar job duties. For instance, within the 2016 construction industry, 62.4% were construction and extraction occupations, 9.7% were office and administrative support occupations, 6.2% were management occupations, and 3.2% were transportation and material moving occupations. (https://www.bls.gov/emp/ep_table_109.htm).

¶¶¶¶ Illicit opioid means heroin or the use of prescription pain relievers in any way not directed by a doctor (does not include over-the-counter medications).

***** https://www.samhsa.gov/data/sites/default/files/NSDUH-DetTabs-2016/NSDUH-DetTabs-2016.pdf.

††††† The Substance Abuse and Mental Health Services Administration (SAMHSA) provides detailed information on drug-free workplace programs, related laws and regulations, and a toolkit for employers (https://www.samhsa.gov/workplace).
